# One page of text: Eye movements during regular and thorough reading, skimming, and spell checking

**DOI:** 10.16910/jemr.11.1.1

**Published:** 2018-02-26

**Authors:** Alexander Strukelj, Diederick C. Niehorster

**Affiliations:** Centre for Languages and Literature, Lund University, Sweden; The Humanities Laboratory, Lund University, Sweden; Department of Psychology, Lund University, Sweden

**Keywords:** Eye movements during reading, eye movement, eye tracking, paragraph reading, reading, reading strategy, thorough reading, saccades, skimming, spell checking, proofreading

## Abstract

Eye movements during regular reading, thorough reading, skimming, and spell checking of single pages of text were measured, to investigate how high-level reading tasks elicited by instructions affect reading behavior. Word frequency and word length effects were found. All results were compared to regular reading. Thorough reading involved longer total reading times and more rereading, and resulted in higher comprehension scores. Skimming involved longer saccades, shorter average fixation durations, more word skipping, shorter total reading times evenly distributed across the page, and resulted in lower comprehension scores. Spell checking involved shorter saccades, longer average fixation durations, less word skipping, longer total reading times evenly distributed across the entire page, and resulted in lower comprehension scores. Replicating local effects shows that paragraphs maintain sufficient experimental rigor, while also enabling reading analyses from a global perspective. Compared to regular reading, thorough reading was more elaborate and less uniform, skimming was faster and more uniform, and spell checking was slower and more uniform.

## Introduction

Research into eye movement characteristics during reading can be said to be almost as old as eye-tracking itself. Huey described the usage of eye-tracking to record eye movements during reading, along with extensive investigations into the underlying mechanisms of reading ( 19 ). In the 1920s, Tinker made seminal studies of how people read text passages of scientific prose, elementary chemistry, algebra, and algebraic formulae ( 47 ). Reading research with eye trackers took flight as high quality, less invasive eye-tracking techniques came about in the 1970s, along with computer systems that allowed for online manipulations of the stimuli (see 34, 35 for overviews). However, the research of eye movements during reading often studies the reading of single sentences. Studies using paragraphs or multiple pages of text are much more infrequent, with many open questions remaining. This has lead researchers to call for more eyetracking research at this more natural level of reading (e.g., 25 ).

In the current study, we present participants with pages of text, along with instructions to read them using four different reading strategies; regular reading, thorough reading, skimming, and spell checking. The aim is to determine the characteristics of thorough reading, skimming, and spell checking, compared to regular reading, and how the differences manifest in eye movement measures. Some previous research has investigated the effects of instruction on reading behavior, but often only contrasting two types of reading, such as regular reading and thorough reading ( 20, 31 ) or regular reading and skimming ( 4, 11 ). This study uses four types of reading, which allows for a more nuanced separation between different reading behaviors, as well as identifying similarities between these types of reading. Contrasting these types of reading provides insight into how high-level cognitive reading tasks are translated into reading behavior, and how the reading process adapts to these different task sets. Different types of reading entail different types of processing and different cognitive demands, which should be evident in eye movements during reading ( 23 ). By investigating the changes in eye movement parameters, we can create a blueprint of eye movement characteristics that can identify each type of reading. Being able to identify each type of reading can provide researchers with new ways of assessing how the reading process reacts to experimental manipulations. This can hopefully help researchers draw more accurate conclusions about reading in general.

During reading, a meaning representation is incrementally created, integrating what is currently read with what has already been read ( 29, 39 ). By using entire paragraphs of text in the current study, we are able to better investigate and provide insight into the process of meaning integration. Research suggests that in order to more fully describe natural reading, sentence processing models should take factors such as semantic plausibility, expectations, and context into account ( 5, 14, 15, 41 ). It could be argued that it is nearly impossible to control and account for all factors believed to influence reading. By using paragraphs rather than single sentences, we can investigate reading under more natural circumstances, thereby drawing more accurate conclusions of how the reading process operates in real life. Research using single sentences is crucial when investigating specific features of the reading process, but should not be used when the investigation of “natural” reading is of interest.

The current study determines eye movement characteristics during the reading of one page of text on a computer screen, and how these characteristics change in response to the different instructions given to the participants. The instructions ask the participants to either read the text regularly, read the text thoroughly, to skim the text, or to spell check the text. Moving beyond single sentences and instead using entire pages of text opens up the possibility of answering questions about text reading strategies, providing a global, paragraph-level perspective to reading research. Global text processing is a subject matter that has been researched before, commonly using whole sentences as the unit of analysis ( 21, 22 ). These studies have argued for the usage of new measures, which have been shown to identify reading styles and literacy skills ( 22, 30 ). As the current study does not focus on reading of sentences, but instead on effects of position in the text on reading behavior, we will adopt line of text as the unit of analysis. In order to establish that sufficient experimental rigor can be maintained, attempts to replicate well-established effects from the sentence processing literature will be performed. This would show that local effects triggered by specific and isolated words are still present when using an entire page of text as stimulus.

## Previous Research

Early eye-tracking measures are used to investigate immediate effects on the reading process. Early measures signify measures that are highly sensitive to largely unconscious effects during reading, which commonly start when a word is fixated for the first time. For instance, first fixation durations have been shown to increase when encountering an infrequent word ( 34 ), and decrease when encountering a predictable word ( 13 ). Another early measure, first-pass reading time, measures the duration of the first entry into a region, and has been shown to increase for syntactically incongruent words ( 38 ). Late measures, on the other hand, signify measures that react to changes during later stages of reading, and commonly include longer periods of reading. Late measures such as total reading times increase with more rereading, and have been shown to increase with more processing ( 31 ), more overall attention ( 20 ), and higher engagement in the text ( 46 ).

It has been shown that reading ability and comprehension can improve by teaching reading strategy, suggesting that reading strategy, and awareness of reading strategy, is very important during thorough reading and learning ( 18 ). Indeed, it is argued that better readers exhibit better metacognitive knowledge compared to poorer reader ( 2 ), meaning that they are better at keeping track of their understanding of the text they are reading.

Skimming can efficiently create an overview of a text under time pressure ( 10 ), and has been shown to have shorter total reading times compared to regular reading ( 45 ). During reading, words are sometimes skipped and the reader does not perform a fixation on the word at all. It has been shown that proficient readers of English text skip around one third of the words ( 7 ). Function words are skipped to a larger extent than content words ( 17 ), and illegal the-previews are skipped to a much larger extent because of parafoveal processing ( 1 ). This is related to the finding that word length influences skipping, with the likelihood of skipping a one- or two-letter word around 76%, a four-letter word around 42%, and a nine- or ten-letters word only around 5% ( 50 ). A study that compared regular reading and scanning text for specific topics, which can be considered similar to skimming, reported that early effects of frequency in first fixation durations and first-pass reading times are similar for these two reading strategies ( 51 ). Another study used a gaze-contingent eye-tracking setup that masked text that had been read, hindering participants from rereading text, simulating speedreading or skimming ( 44 ). The study found that comprehension decreased, concluding that comprehension is indeed supported by regressive eye movements. The study questions the viability of speedreading, which could be seen as a process similar to skimming the text. This is also argued by Rayner, Schotter, Masson, Potter, and Treiman ( 37 ), who suggest that the only way of increasing reading speed while maintaining high comprehension is to increase language skill. The measure number of visits on a word can show skipping from a global perspective.

Normally, studies use the term proofreading when participants search for spelling errors. However, this term is problematic, as proofreading can also signify searching for other mistakes such as subject-verb agreement errors, logical mistakes, and possibly even incorrect facts. The term spell checking is therefore used in the current study. A study investigated spell checking by giving people sentences without spelling errors to be read for comprehension, followed by sentences with an occasional spelling error ( 27 ). In this second group of sentences, instructions specified that spelling errors might occur, and that the participant should indicate this after the sentence. Many differences in eye movements were observed. For instance, saccade amplitudes (the length of a saccade in pixels) were shorter and refixation probabilities were higher during spell checking. Moreover, both the word length and word frequency effects in first-pass reading times were greater during spell checking. The study concluded that attentional resources are significantly modulated by task effects. The increased frequency effect for spell checking was replicated in another study, further suggesting that readers change their reading strategies depending on the task demands ( 43 ). It is possible that spell checking will increase fixation durations compared to regular reading because of the need for higher amounts of activation in order to verify the correct spelling of each word.

## The Current Study

This study used four different types of instructions to make participants read a text in a certain manner. These types of reading were chosen to investigate four commonly used types of reading, namely regular reading, thorough reading, skimming, and spell checking. Regular reading was used as the baseline condition and compared to the remaining three types. Previous research, as described above, has shown differences in these types of reading, which make them very interesting to investigate and contrast in depth using eye tracking. The task in regular and thorough reading can be seen as studying the content and information of the text, while the task in spell checking can be seen as investigating the details of the individual words. The task in skimming can be seen to quickly get an overview of the information but not dwell on the specifics of the text. Specific reading styles have been identified when performing cluster analyses of eye movement patterns in long expository text ( 21, 24 ). This shows that reading styles can be identified in eye movement measures during paragraph reading. Repeated reading has been shown to facilitate processing ( 23 ), possibly because it is less cognitively demanding to integrate new information into an already existing meaning representation, than when the meaning representation is less complete ( 29, 39 ). In Hyönä and Niemi ( 23 ), this was seen in shorter total reading times. It is possible that these effects could be seen in different eye movement characteristics in the beginning and ends of texts. Accordingly, Hyönä and Niemi ( 23 ) showed that average fixation durations got shorter as text was read, ending with the shortest average fixation durations on sentences in the conclusions.

### Terms and Classifications

Four different types of reading were used in this study, namely regular reading, thorough reading, skimming, and spell checking. Below follows a short description of how the terms are understood in this study.

The term regular reading signifies what is commonly referred to as reading for comprehension in previous research ( 35, 43, 49 ), where participants are instructed to read the text as they would read the text normally ( 16, 28 ). This type of reading is used as the baseline condition, which the remaining three types of reading will be compared to.

The term thorough reading signifies what is referred to as reading to learn ( 40 ) in previous research, where participants are instructed to read the text thoroughly in order to learn the material in it ( 46 ), or being told they should learn the material as there is a test afterwards ( 12 ). When participants are thoroughly reading a text, it can be assumed that the comprehension and most importantly retention of the material will be higher than regular reading, and that the total time spent on the text will be higher than regular reading.

The term skimming signifies what is referred to as reading for gist ( 35 ) in previous research, that is, trying to read a text as fast as possible while still understanding it ( 10, 37, 52 ). When participants are skimming a text, it can be assumed that the comprehension and most importantly retention of the material will be lower than regular reading, and that the total time spent on the text will be lower than regular reading.

The term spell checking signifies the way participants read when they try to find words that are misspelled, something that is often referred to as proofreading in previous research ( 27, 44 ), where participants are instructed to pay attention “to the letters and the spelling of the words without paying attention to the meaning” ( 7 ) and “are expected to attend strongly to the orthography of words” ( 53 ). When participants are spell checking a text, it can be assumed that the comprehension and retention of the material will be lower than regular reading, and that the total time spent on the text will be higher than during regular reading.

### Hypotheses

Several hypotheses were formed for the differences between regular reading and the remaining the three types of reading. A summary of the hypotheses and the hypothesized effects are given in Table 1.

**Table 1.** Hypotheses and hypothesized effects on the experimental measures compared to regular reading.

	Hypothesis compared to regular reading	Hypothesized effects compared to regular reading
Thorough reading	Better comprehension of material	Higher comprehension scores
	No difference in processing on words for all fixations	Average fixation durations similar to regular reading
	No difference in distances between two subsequent fixations during reading	Saccade amplitudes similar to regular reading
	More rereading of previously read text	Higher proportion of vertical saccades
	More regressions	Higher proportion of leftward saccades
	More deliberate reading during entire trial	Longer total reading times, higher number of visits on words
	Reading is very similar to regular reading when investigating one entire trial	Reading pattern is similar to regular reading during entire trial
Skimming	Reduced comprehension of material	Lower comprehension scores
	Lower amounts of processing on words for all fixations	Shorter average fixation durations
	Larger distances between two subsequent fixations during reading	Larger saccade amplitudes
	Less rereading of previously read text	Lower proportion of vertical saccades
	Fewer regressions	Lower proportion of leftward saccades
	Less deliberate reading during entire trial	Shorter total reading times, lower number of visits on words
	Uniform reading pattern across the entire text	Total reading times and number of visits are similar over the entire text
Spell checking	Reduced comprehension of material	Lower comprehension scores
	Higher amounts of processing on words for all fixations	Longer average fixation durations
	Smaller distances between two subsequent fixations during reading	Smaller saccade amplitudes
	More pronounced frequency effect	Even longer first fixation durations and first-pass reading times for less frequent compared to more frequent words
	More pronounced word length effect	Even longer total reading times for longer compared to shorter words
	No differences in rereading of previously read text	Proportion of vertical saccades similar to regular reading
	More regressions	Higher proportion of leftward saccades
	More deliberate reading during entire trial	Longer total reading times, higher number of visits on words
	Uniform reading pattern across the entire text	Total reading times and number of visits are similar over the entire text

During thorough reading, better comprehension is expected as participants engage thoroughly with the material, shown by higher comprehension scores. A larger distance between two subsequent fixations, a larger saccade amplitude, indicates a larger frequency of skips during reading. This is not expected during thorough reading, but more rereading of previously read text up and down the page is hypothesized, shown by a higher proportion of vertical saccades. Also, a higher proportion of leftward, i.e., regressive, saccades, indicating a higher number of regressions, are expected, as this could indicate thorough reading of individual lines of text. Processing as defined by average fixation durations indicate higher processing during every fixation. This is not expected for thorough reading, but more time is expected on each word during the entire trial, shown by increases in total reading times per character and total number of visits. The pattern of reading one page of text is expected to be similar to regular reading, shown by longer total reading times per character and a higher total number of visits on words early in the text compared to the words toward the end. Participants are expected to spend more time in total on the beginning of the text compared to the end of the text, which is also the case in regular reading, but all durations are expected to be longer than regular reading.

During skimming, worse comprehension is expected as participants only form an overview of the material, shown by lower comprehension scores. Processing should be lower on each word during every fixation, overall reading should be faster and more spread out over the page, and more word skips should be found, shown by shorter average fixation durations and longer saccade amplitudes. A lower proportion of vertical saccades is expected, indicating less rereading of previously read text up and down the page, and a lower proportion of leftward i.e., regressive, saccades, indicating a lower number of regressions. Less time should be spent on each word during the entire trial, shown by decreases in total reading times per character and total number of visits. The pattern of reading one page of text is expected to be uniform regardless of which word is fixated, shown by similar total reading times per character and similar total number of visits regardless of the location of the words in the text. In other words, eye movement characteristics are expected to be manifested in a uniform manner during reading, irrespective of where in the text a word is situated. Participants are expected to only form an overview of the material and not using more processing than needed.

Spell checking is more similar to a search task than reading. This should produce lower comprehension scores, as participants are not reading the text in order to understand the content, but are instead searching for errors. Average fixation durations should be longer and saccade amplitudes should be shorter, as participants perform eye movements that are slower, less spread out over the page, and skip fewer words during reading. The frequency effect on first fixation durations and first-pass reading times is expected to be larger, as participants are less certain of the spelling of infrequent words and thus need more processing when initially encountering infrequent words. The effect of word length on total reading times is expected to be larger, as long words contain more letters and therefore more possibilities for spelling errors. Total reading times per character and total number of visits should increase. The pattern of reading one page of text is expected to be uniform regardless of which word is fixated, shown by similar total reading times per character and similar total number of visits regardless of the location of the words in the text. Participants are expected to search for errors rather than reading regularly.

## Methods

### Participants

Sixty-four native speakers of Swedish (34 female; 18 to 29 years of age) participated in the experiment. The participants were recruited through student lists and advertisements at the Lund University campus. All participants had normal or corrected-to-normal vision. Four participants were excluded from the results due to technical problems with the eye-tracking equipment or poor recordings. This resulted in 60 participants in total (32 female; M=22.9 years, SD=3.69). The participants were naïve to the purpose of the experiment.

### Apparatus

Eye movement data were recorded binocularly at a sampling rate of 120 Hz using RED-m remote videobased eye-trackers (SensoMotoric Instruments, Teltow, Germany). The experiment took place in the Digital Classroom at the Humanities Laboratory, Lund University, Sweden. The distance from a participant’s eyes to the stimulus monitor was approximately 60 cm. Stimuli were displayed on a Dell P2210 22" widescreen LCD display with a refresh rate of 60 Hz at a resolution of 1,680 × 1,050 pixels (47.5 × 30.0 cm, 43.2 × 28.1 degrees of visual angle at a distance of 60 cm). The eye-tracking system was controlled by SMI iView RED-m (3.2.177), while stimuli presentation, a 5-point calibration, and a 4point validation of the calibration accuracy were controlled by SMI Experiment Center (3.5.281).

### Design and Stimuli

The stimuli consisted of eight texts taken from the International Reading Speed Texts (IReST) ( 48 ). One text was also used as an example stimulus. The IReST texts are balanced for text length, word length, and linguistic complexity. The IReST texts are available in several languages, and the Swedish version was used. All Swedish texts had 146 words and 16 lines, 684 characters in total, with an identical readability index (LIX 35). The readability index approximately corresponds to the readability of fiction books. The mean word length is 4.61 (SD=0.01) characters, and is balanced across the texts. Each text was adapted to on-screen reading by using a monospaced ClearType font, namely Consolas at a 26 point size (approximately 4.5 × 6.5 mm, 0.35 × 0.50 degrees of visual angle at a distance of 60 cm).

**Figure 1.**
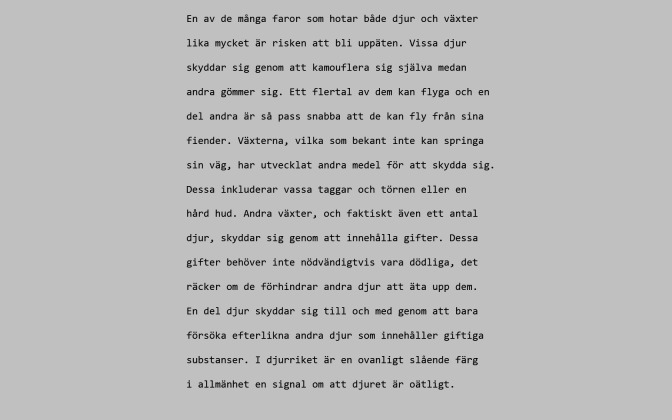
Example stimulus screen.

Each individual line of text was separated by one blank line in order to facilitate accurate eye-tracking measures. An example stimulus screen can be found in Figure 1.

The original English version of the text can be found in Appendix 1. The translations were verified to correspond accurately with the original texts. None of the texts contained any spelling errors, even though one of the instructions were to inspect a text for spelling errors. Adding spelling errors into the text was considered, but in order to compare identical texts, this was not done. Each text was followed by five multiple-choice questions. Each question had five alternatives, with only one alternative being correct. An example question for the text in Figure 3.1 can be found in (1) below, translated into English. The questions were given in Swedish during the experiment.

(1) What is described as a danger for animals and plants alike?

A. To be killed.

B. To be poisoned.

C. To be eaten.

D. To be injured.

E. None of the above.

The texts were distributed over four lists using a Latin square design, and the order of appearance of the texts was randomized. The lists contained two texts in each one of the four conditions, namely thorough reading, skimming, spell checking, and regular reading (which was the baseline condition). The distribution was counterbalanced using a Latin square design so that the instructions were paired with different texts for each participant. For regular reading, the written instructions were to read the text so the participant understood it. For thorough reading, the written instructions were to read the text very thoroughly. For skimming, the written instructions were to “read the text quickly, that is, skim the text”, but to try to still understand it. For spell checking, the written instructions were to look for spelling errors in the text. The texts in the spell checking condition were accompanied with a question regarding the number of spelling errors found.

### Procedure

The experimental session lasted around 30 minutes, but no time restrictions were imposed during the experiment. The participants were greeted and told to sit at specific places in the Digital Classroom, so that they would not see the screens of any other participants. Participant groups ranged from one to five, with three being the most common. They were first informed that their participation was voluntary and that they could stop the experiment at any time. They were then told that the study was a reading study where they would read texts and answer five multiple-choice comprehension questions afterwards, and that they would be given four different instructions prior to each text. They were specifically told that it was very important that these instructions were followed. The participants were given thorough descriptions of each instruction, and were specifically told that it was very important that these instructions were followed during each trial.

They were then seated in front of the eye tracker and a calibration was started. The calibration was repeated until the offset was less than 1.0 degrees of visual angle in both the horizontal and the vertical direction. The experiment started with the participant completing a questionnaire about their age and gender. They were then given an example trial, using the baseline condition instructions of regular reading. The text was also taken from the IReST set of texts, and was accompanied with five questions. After the example trial, they were instructed to call for the experimenter if anything remained unclear, otherwise the experiment would commence. The eye-tracking portion then started, with instructions being shown before each text, and five multiple-choice comprehension questions after each text.

After the eye-tracking portion of the experiment was completed, they were taken outside the Digital Classroom and told about the specifics of the study, signed a consent form, thanked again, and given a movie ticket as compensation for participating in the study.

### Data Analysis

Velocity-based high-speed event detection was performed using SMI BeGaze 3.5 with default settings (peak velocity threshold=40°/s, minimum fixation duration=50 ms), which transformed the raw data into fixations and saccades. The recordings from the right eye were used as the right eye is dominant for the majority of people. Fixations above 1000 ms were removed.

The data processing, statistical analyses, and plots were made using R (3.2.2) ( 33 ) and the lme4 package (1.1-10) in R Studio (0.99.473). Models used random intercepts and slopes of condition within group for participant and item, with intercept-slope correlation factors for participants and items. In the cases where models did not converge, the intercept-slope correlation factors were dropped. This was done on the models for first fixation durations and first-pass reading times as a function of word frequency, and first fixation durations and first-pass reading times as a function of word length. All statistical models use log-transformed data to better fit a Gaussian distribution except where indicated. In order to improve readability, no log-transformation was performed on the data in the figures, except for saccade amplitudes. The lmerTest package (2.0-29) was used to estimate *p*-values with Satterthwaite approximation.

In all saccade analyses, all *return sweeps* (the saccade from the end of one line of text to the beginning of the next line) that were part of initial reading were removed from the results by excluding all saccades directed downward and right-to-left that were over 500 pixels in horizontal width (the width of one line of text was around 700-800 pixels) and under 100 pixels in vertical width (the distance between the middle of two lines of text was around 60 pixels). This resulted in the removal of 5580 saccades from the data (from a total of 93865). Saccades of larger vertical widths and/or smaller horizontal widths were considered part of patterns of rereading rather than the regularity of initial reading patterns. The thresholds were chosen in order to under-remove rather than overremove data. Without the large return sweeps, the analyses of saccade amplitudes should be more accurate, and the analyses of saccade directions should better reflect the special characteristics of each type of reading without the return sweeps that are common to all types and adds unnecessary noise to the data.

The Swedish corpus Korp (https://spraakbanken.gu.se/korp/) , consisting of around 9 million words, was used when investigating frequency effects.

## Results

Full statistical model outputs that are not given in the corresponding results section below can be found in the Online Supplementary Materials. All models except comprehension scores and total number of visits on words use log-transformed data to better fit a Gaussian distribution.

### Comprehension Scores

**Figure 2.**
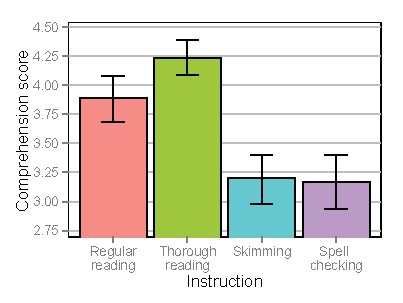
Mean comprehension scores depending on instruction. Error bars denote standard errors of the mean.

Figure 2 shows the comprehension scores. When comparing with regular reading, a significant increase was found for the comprehension scores in the condition with thorough reading (Estimate=0.349, SE=0.135, t=2.59, p<.02), and a significant decrease was found in the remaining two conditions (Skimming: Estimate=0.687, SE=0.165, t=-4.16, p<.001; Spell checking: Estimate=-0.719, SE=0.152, t=-4.72, p<.001). Each text was accompanied by five questions, for a maximum of five points per text.

### Trial Duration

**Figure 3.**
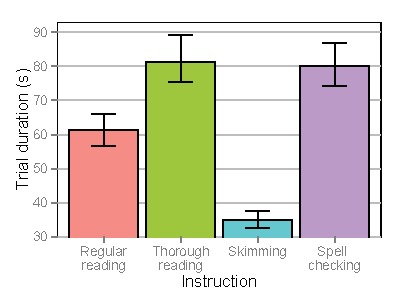
Trial duration depending on instruction. Error bars denote standard errors of the mean.

Figure 3 shows trial duration, i.e., the total time a text was shown on screen before the participant moved on to the comprehension questions. When analyzing trial durations, a significant decrease was found for skimming (Estimate=-0.560, SE=0.052, t=-10.80, p<.0001) and significant increases were found for thorough reading (Estimate=0.275, SE=0.030, t=9.22, p<.0001) and spell checking (Estimate=0.253, SE=0.067, t=3.77, p<.001), when comparing to regular reading.

### Fixations and Saccades

**Figure 4.**
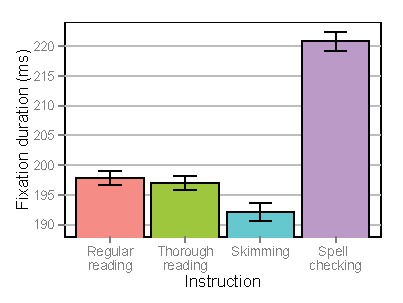
Average fixation durations depending on instruction. Error bars denote standard errors of the mean.

**Figure 5.**
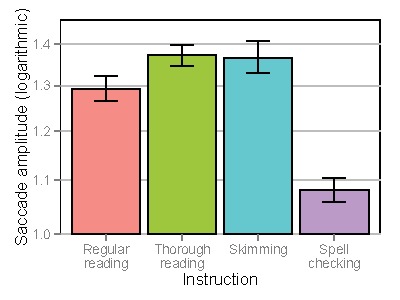
Mean saccade amplitudes (logarithmic scale) depending on instruction (return sweeps are removed). Error bars denote standard errors of the mean.

Figure 4 shows average fixation durations and Figure 5 shows saccade amplitudes. When analyzing average fixation durations, a significant decrease was found for skimming (Estimate=-0.031, SE=0.011, t=-2.74, p<.01) and a significant increase was found for spell checking (Estimate=0.084, SE=0.015, t=5.60, p<.0001), when comparing to regular reading. No differences were found for thorough reading (Estimate=-0.005, SE=0.010, t=0.51, p=.62). When analyzing saccade amplitudes, a significant increase was found for thorough reading (Estimate=0.059, SE=0.015, t=3.92, p<.0001) and skimming (Estimate=0.090, SE=0.018, t=4.92, p<.0001) and a significant decrease was found for spell checking (Estimate=-0.167, SE=0.015, t=-10.90, p<.0001), when comparing to regular reading.

Table 2a shows proportions of vertical saccades and Table 2b shows proportions of leftward saccades. When analyzing proportions of vertical saccades, significant increases were found for thorough reading (Estimate=0.063, SE=0.026, t=2.44, p<.01) and spell checking (Estimate=0.149, SE=0.026, t=5.77, p<.0001), and a significant decrease was found for skimming (Estimate=0.176, SE=0.033, t=-5.38, p<.0001), compared to regular reading. When analyzing proportions of leftward, i.e., regressive, saccades, significant increases were found for spell checking (Estimate=0.063, SE=0.026, t=2.44, p<.01), and significant decreases were found for skimming (Estimate=-0.123, SE=0.026, t=-4.68, p<.0001), compared to regular reading. No significant results were found for thorough reading (Estimate=0.016, SE=0.020, t=0.82, p=.41).

**Table 2a.** Mean proportions of vertical saccades and percentage point (PP) differences from baseline condition (return sweeps are removed). Statistically significant results in bold.

	*Vertical saccades*
	%	PP +/-
Regular reading	14.92	-
Thorough reading	15.53*	0.61*
Skimming	12.79***	-2.13***
Spell checking	16.94***	2.02***

Note: * indicates that the binomial generalized linear mixed model reported *p*<.05 for the comparison with regular reading, *** indicates *p*<.001.

**Table 2b.** Mean proportions of leftward saccades and percentage point (PP) differences from baseline condition (return sweeps are removed). Statistically significant results in bold.

	*Leftward saccades*
	%	PP +/-
Regular reading	23.37	-
Thorough reading	24.17	0.80
Skimming	21.25***	-2.02***
Spell checking	24.45*	1.08*

Note: * indicates that the binomial generalized linear mixed model reported *p*<.05 for the comparison with regular reading, *** indicates *p*<.001.

**Figure 6.**
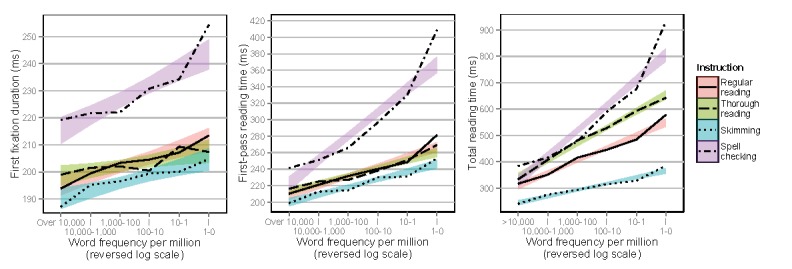
First fixation durations, first-pass reading times, and total reading times on words depending on instruction as a function of word frequency per million, using a log-scale running from high to low, separated by reading type.

Figure 6 shows first fixation durations, first-pass reading times, and total reading times on words as a function of word frequency. The frequency effect for first fixation durations, well-known from previous sentence reading research ( 3, 36 ), was replicated, with first fixation durations increasing as a function of decreasing frequency (Estimate=0.014, SE=0.003, t=4.67, p<.0001). Overall, first fixation durations were also significantly longer for spell checking (Estimate=0.075, SE=0.015, t=4.84, p<.0001), compared to regular reading. No other significant results were found (all ps>.15). This effect was also found for first-pass reading times, which increased as a function of decreasing frequency (Estimate=0.040, SE=0.003, t=11.7, p<.0001). Overall, first-pass reading times were also significantly longer for spell checking (Estimate=0.075, SE=0.015, t=4.84, p<.0001), compared to regular reading. An interaction was also found between spell checking and frequency (Estimate=0.024, SE=0.005, t=5.02, p<.0001), showing that the frequency effect was larger for spell checking than for regular reading. This effect was also found for total reading times, which increased as a function of decreasing frequency (Estimate=0.101, SE=0.005, t=21.7, p<.0001). All conditions and all interactions were also significantly different compared to regular reading (for condition and interaction data, see the full statistical model outputs in the Online Supplementary Materials).

**Figure 7.**
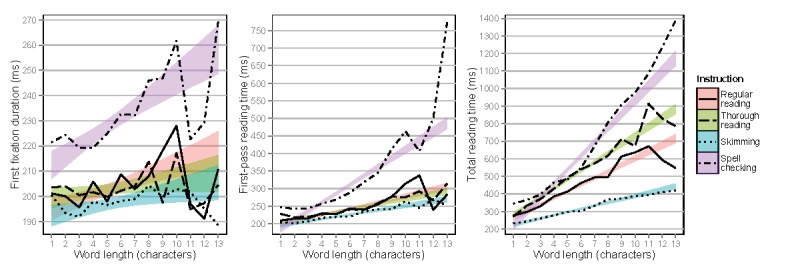
First fixation durations, first-pass reading times, and total reading times on words depending on instruction as a function of word length, separated by reading type. Means are shown by lines, only 95% confidence intervals are shown for regression lines.

**Figure 8.**
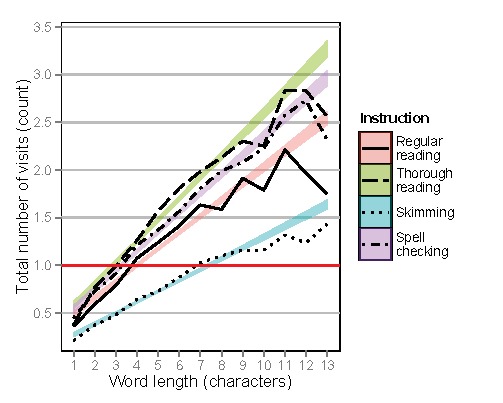
Total number of visits on words depending on instruction as a function of word length, separated by reading type. Means are shown by lines, only 95% confidence intervals are shown for regression lines.

Figure 7 shows first fixation durations, first-pass reading times, and total reading times on words as a function of word length. Figure 8 shows total number of visits as a function of word length. The word length effect, well-established by previous sentence reading research( 34, 35 ), was replicated, with significant increases as a function of word length for first fixation durations (Estimate=0.009, SE=0.002, t=4.29, p<.0001), first-pass reading times (Estimate=0.030, SE=0.002, t=13.6, p<.0001), total reading times (Estimate=0.078, SE=0.003, t=26.4, p<.0001), and number of visits (Estimate=0.171, SE=0.004, t=42.6, p<.0001). When compared to regular reading, spell checking showed longer first fixation durations (Estimate=0.061, SE=0.016, t=3.92, p<.0001), and skimming showed shorter total reading times (Estimate=-0.144, SE=0.040, t=-3.63, p<.0001) and fewer number of visits (Estimate=-0.166, SE=0.070, t=-2.36, p<.03). Significant interactions with word length were also found, when compared to regular reading. First-pass reading times increased for spell checking (Estimate=0.023, SE=0.003, t=7.44, p<.0001), and both total reading times and number of visits increased for thorough reading and spell checking, but decreased for skimming (for interaction data, see the full statistical model outputs in the Online Supplementary Materials).

In the subsequent analyses in this study, interesting results were found when using the position of the word or line in the text. However, the results found for word frequency or word length should be unaffected by this, as the IReST stimuli texts are balanced for word length, lexical complexity, number of words, and number of lines.

### Total Reading Times per Character for Lines of Text and for Individual Words

Figure 9a shows total reading times per character for lines of text and Figure 9b shows total reading times per character for individual words (excluding skipped words) depending on reading type. When analyzing total reading times per character for lines of text, significant increases were found for thorough reading (Estimate=0.226, SE=0.062, t=3.66, p<.01) and spell checking (Estimate=0.301, SE=0.076, t=3.97, p<.001), and significant decreases were found for skimming (Estimate=-0.496, SE=0.072, t=-6.87, p<.0001), when compared with regular reading. When using individual words, all skipped words were removed from the analyses and figures. As when using lines of text, significant increases were found for thorough reading (Estimate=0.145, SE=0.029, t=4.94, p<.0001) and spell checking (Estimate=0.188, SE=0.046, t=4.09, p<.0005), and significant decreases were found for skimming (Estimate=-0.288, SE=0.030, t=-9.58, p<.0001), when compared with regular reading.

**Figure 9a.**
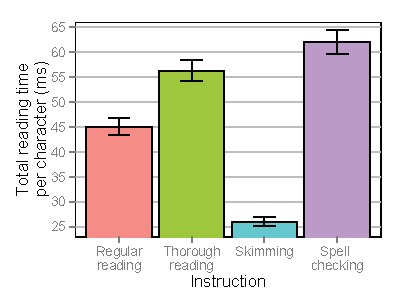
Total reading times per character for lines of text depending on instruction. Error bars denote standard errors of the mean.

**Figure 9b.**
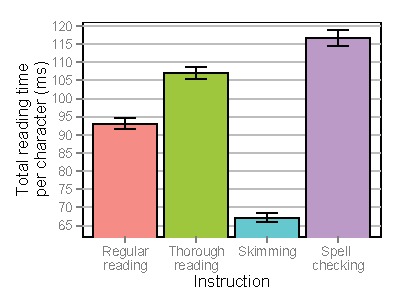
Total reading times per character for individual words (excluding skipped words) depending on instruction. Error bars denote standard errors of the mean.

**Figure 10.**
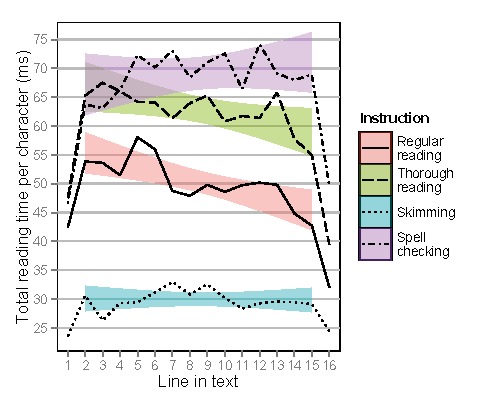
Total reading times per character for lines of text depending on instruction as a function of line order in the text. Means are shown by lines, 95% confidence intervals are shown for regression lines when excluding first and last lines.

Figure 10 shows total reading times per character on individual lines, on each individual line, along with a regression line for all lines except the first and last. This was done because the first and last lines were found to differ greatly from the rest of the data. All subsequent analyses and figures were made without the words in the first and last line of text. When using the relative position of the individual lines, total reading times per character were longer as a function of line number for spell checking only, compared to regular reading. The full statistical model output can be found in Table 3.

**Table 3.** Full statistical model output: Total reading times per character for lines of text as a function of line order in the text, excluding the first and last lines. Statistically significant results in bold. A colon denotes an interaction.

		Estimate	SE	df	*t*-value	*p*-value	
*Thorough reading*		**0.2334**	**0.0729**	**100**	**3.202**	**.00183**	**
*Skimming*		**-0.6218**	**0.0868**	**49**	**-7.162**	**.00000**	***
*Spell checking*		**0.1789**	**0.0879**	**75**	**2.034**	**.04550**	*
*Line number*		0.0037	0.0042	6415	0.865	.38717	
*Thorough reading: Line number*		0.0003	0.0060	6422	0.047	.96261	
*Skimming: Line number*		0.0117	0.0061	6431	1.920	.05489	.
*Spell checking: Line number*		**0.0148**	**0.0060**	**6413**	**2.472**	**.01347**	*

**Figure 11.**
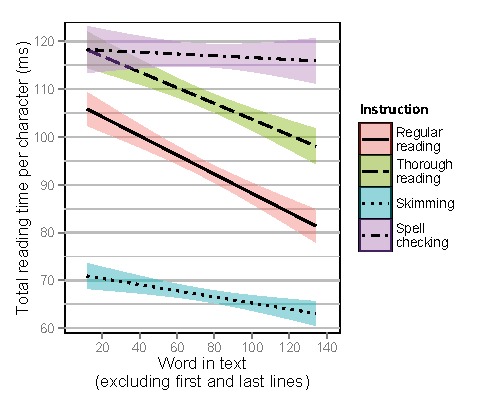
Total reading times per character for words as a function of word order in the text, excluding skipped words, without first and last lines, depending on instruction. Regression lines include 95% confidence interval.

Figure 11 shows regression lines for total reading times per character for words as a function of the relative position of each word in the text, excluding skipped words and the words in the first and last lines. When using the relative position of the words in the text, total reading times per character decreased less as a function of word number for skimming and spell checking, compared to regular reading. The full statistical model output can be found in Table 4.

**Table 4.** Full statistical model output: Total reading times per character for words as a function of word order in the text, when excluding skipped words, without first and last lines. Statistically significant results in bold. A colon denotes an interaction.

		Estimate	SE	df	*t*-value	*p*-value	
*Thorough reading*		**0.1181**	**0.0390**	**35**	**3.026**	**.00461**	**
*Skimming*		**-0.3571**	**0.0394**	**74**	**-9.050**	**.00000**	***
*Spell checking*		0.06.686	0.0550	33	1.215	.23317	
*Word number*		**-0.0011**	**0.0002**	**36490**	**-5.249**	**.00000**	***
*Thorough reading: Word number*		0.0004	0.0003	36440	1.511	.13082	
*Skimming: Word number*		**0.0008**	**0.0003**	**36120**	**2.532**	**.01134**	*
*Spell checking: Word number*		**0.0016**	**0.0003**	**36720**	**5.583**	**.00000**	***

### Total Number of Visits

**Figure 12.**
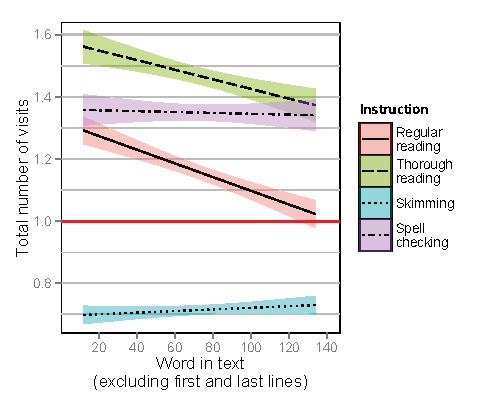
Total number of visits on words as a function of word order in the text, without first and last lines, depending on instruction. Regression lines include 95% confidence interval.

Figure 12 shows regression lines for total number of visits on words as a function of the relative position of each word in the text, excluding the words in the first and last lines. When analyzing the total number of visits on individual words, they were found to be higher as a function of word number for skimming and spell checking, compared to regular reading. These results indicate that the decrease found for regular reading is not present for skimming and spell checking. The full statistical model output can be found in Table 5.

**Table 5.** Full statistical model output: Total number of visits on words as a function of word order in the text, excluding first and last lines. Statistically significant results in bold. A colon denotes an interaction.

		Estimate	SE	df	*t*-value	*p*-value	
*Thorough reading*		**0.2536**	**0.0755**	**26**	**3.358**	**.00247**	**
*Skimming*		**-0.6153**	**0.0730**	**34**	**-8.431**	**.00000**	***
*Spell checking*		0.0661	0.0986	37	0.671	.50675	
*Word number*		**-0.0021**	**0.0003**	**59640**	**-7.460**	.00000	***
*Thorough reading: Word number*		0.0007	0.0004	59640	1.780	.07502	.
*Skimming: Word number*		**0.0024**	**0.0004**	**59640**	**5.812**	**.00000**	***
*Spell checking: Word number*		**0.0018**	**0.0004**	**59640**	**4.362**	**.00001**	***

## Discussion

This eye-tracking study investigated the eye movement characteristics of different types of reading, using an entire page of text instead of single sentences. Both entire lines of text and isolated words were used in the analyses. Participants were reminded of the importance of using the indicated reading strategy, both during the verbal instructions before the experiment and the written instructions before each trial. To verify that participants were actually performing the instructed reading, the results from the comprehension questions were used. Significant increases in comprehension scores were found for thorough reading, and significant decreases for skimming and spell checking, compared to regular reading. Also, trial durations (the total time a text was present on screen before the participant continued to the comprehension questions) were significantly shorter for skimming, and significantly longer for thorough reading and spell checking, as would be expected. This suggests that participants followed the instructions, and that the eye-tracking data should thus reflect the reading characteristics for the different reading types.

The word frequency effect found in previous eyetracking research was replicated ( 3, 36 ), finding significant increases in first fixation durations and first-pass reading times as a function of decreasing frequency. The effect for word length found in previous eye-tracking research was also replicated ( 34, 35 ), with significant increases in first fixation durations, first-pass reading times, total reading times, and total number of visits as a function of word length. Replicating the frequency and word length effects using a page of text shows that local effects are still evident in the data, despite using whole paragraphs of text instead of single sentences as is done in this previous work. Using an entire page of text as stimuli offers the additional advantage of allowing the analysis of reading from a global perspective, answering questions about the reading process when it operates in a more natural way.

In this study, rather than using skipping rate or total number of refixations, the total number of visits was used, as a global measure examining rereading operating on an entire page of text. Word skipping has been shown to increase for short words ( 50 ), function words ( 17 ), illegal the-previews ( 1 ), and it is argued that proficient readers of English normally skip around one third of the words ( 7 ). In this study, examining the number of visits to a words throughout the text revealed that skipping also depends on the word’s location in the text. Thus, the total number of visits to a word was used to show general reading strategies rather than specific skipping effects.

Each section below begins with replications of previous research on different reading types, followed by the additional conclusions that can be drawn from the data in this study. Table 6 shows a summary of the behavior and measured effects, and how they correlate with the hypotheses and hypothesized effects presented in the section The Current Study above.

**Table 6.** Behavior and measured effects on the experimental measures compared to regular reading. Hypotheses and effects supported by the data in bold.

	Behavior compared to regular reading	Measured effects compared to regular reading
Thorough reading	**Better comprehension of material**	**Higher comprehension scores**
	No difference in processing on words for all fixations	Average fixation durations similar to regular reading
	Larger distances between two subsequent fixations during reading	Larger saccade amplitudes similar to regular reading
	**More rereading of previously read text**	**Higher proportion of vertical saccades**
	No difference in regressions on the current line of text	No difference in proportion of leftward saccades
	**More deliberate reading during entire trial**	**Longer total reading times, higher number of visits on words**
	**Reading behavior is similar to regular reading when investigating fixations durations on words in one entire trial**	**Reading pattern is similar to regular reading during entire trial**
Skimming	**Worse comprehension of material**	**Lower comprehension scores**
	**Lower amounts of processing on words for all fixations**	**Shorter average fixation durations**
	**Larger distances between two subsequent fixations during reading**	**Larger saccade amplitudes **
	**Less rereading of previously read text**	**Lower proportion of vertical saccades**
	**Fewer regressions**	**Lower proportion of leftward saccades**
	**Less deliberate reading during entire trial**	**Shorter total reading times, lower number of visits on words**
	**Uniform reading pattern across the entire text**	**Total reading times and number of visits are similar over the entire text**
Spell checking	**Worse comprehension of material**	**Lower comprehension scores**
	**Higher amounts of processing on words for all fixations **	**Longer average fixation durations**
	**Smaller distances between two subsequent fixations during reading**	**Smaller saccade amplitudes **
	**More pronounced frequency effect **	**Even longer first fixation durations and first-pass reading times for less frequent compared to more frequent words**
	**More pronounced word length effect**	**Even longer total reading times for longer compared to shorter words**
	More rereading of previously read text	Higher proportion of vertical saccades
	**More regressions **	**Higher proportion of leftward saccades **
	**More deliberate reading during entire trial**	**Longer total reading times, higher number of visits on words**
	**Uniform reading pattern across the entire text**	**Total reading times and number of visits are similar over the entire text**

### Thorough Reading

Trial durations were significantly longer, compared to regular reading. This means that people spent more time reading the texts before moving to the next one.

*Replication of previous research.* Thorough reading had significantly longer total reading times per character, compared to regular reading. As comprehension also increased, shown in higher comprehension scores, this suggests a link between learning and eye movements, replicating previous research in multimedia learning ( 6, 8, 26, 32 ). Longer total reading time can show more processing ( 31 ), more engagement ( 46 ), and more attention ( 20 ), which can all be said to reflect thorough reading.

*Discussion of new findings.* When comparing the reading profile to regular reading, the profiles are very similar. No differences were expected for saccade amplitudes, but they were significantly larger than regular reading, with average fixation durations not significantly different. The increases in saccade amplitudes might be explained by significantly longer trials in combination with the higher proportion of vertical saccades, which are long regressive saccades to much earlier parts of the text. As these are fairly frequent, it affected the average saccade amplitudes. The finding that the proportion of leftward, i.e., regressive, saccades was not significantly larger shows that rereading was not primarily of the current line of text but rather of earlier material. This suggests that overall reading strategies were fairly similar to regular reading, but with more rereading of previously read text rather than more frequent rereading on the current line. Leftward, regressive saccades are made to reread the information currently being processed. Regressive saccades are important, as completely blocking the ability to reread previously read text has been shown to negatively affect comprehension ( 44 ). However, regressive saccades seem to play a minor role in increasing comprehension, with vertical saccades being much more important when increasing comprehension and possibly learning. Vertical saccades can be seen as global rereading focused on “filling in the blanks” of knowledge. This potentially reflects a reading strategy that leads to better comprehension ( 2, 18 ). Furthermore, total reading times were longer but not significantly different over the page. The downward slope found in total reading times from the beginning to the end of the text was similar to the one found for regular reading. This slope, combined with the higher proportion of vertical saccades, shows the rereading behavior of previously read material. As a result, reading of the initial parts of the text was more thorough when considering reading during the entire trial. It is likely that the participant needs to recheck a detail that was forgotten, or perhaps establishing a context for the rest of the text. It is also possible that the text is reread to a larger extent simply because the text is available for rereading for a longer time. It is possible that the pattern reflects high demands of integrating new information and building a meaning representation of the text during initial stages of reading, when this representation is very sparse ( 29, 39 ). These demands decrease as the memory representation becomes more complete during later stages of reading. This is similar to how total reading times become shorter after repeated reading and how average fixation durations become shorter as more of a text is read ( 23 ).

Total reading times per character were the longest for spell checking, both overall and as a function of word length, but very long for thorough reading as well. However, the total number of visits on words as a function of word length were the highest for thorough reading. Combined, this suggests that words were reread and revisited to a larger extent during thorough reading, and that the results for spell checking signify increased processing and less rereading. This is also found in the reading pattern for total number of visits, which exhibits the same downward slope as for total reading times for thorough reading, and an even pattern across the entire page for spell checking.

### Skimming

Trial durations were significantly shorter, compared to regular reading. This means that people spent less time reading the texts before moving to the next one.

*Replication of previous research.* Skimming had significantly longer saccade amplitudes, compared to regular reading. This replicates the theory described for the realtime reading-skimming classifier in Biedert et al. ( 4 ). Total reading times per character were significantly shorter, compared to regular reading. This replicates the results in Simola et al. ( 45 ) and Duggan and Payne ( 10 ). The amount of rereading was very low compared to regular reading, with significantly smaller proportions of vertical and leftward, i.e., regressive, saccades. The correlation between less rereading and lower comprehension scores replicates Schotter, Tran, et al.( 44 ), where participants were not able to reread text. Frequency effects in first fixation durations and first-pass reading times consistent with regular reading were found, replicating ( 51 ).

*Discussion of new findings.* With regards to the reading pattern across the page, similarities can be found with spell checking, with both total reading times and total number of visits being very even across the entire text (albeit much longer and higher for spell checking and much shorter and lower for skimming.) These results show that, compared to regular reading, processing was lower but more evenly distributed across the entire page of text, longer saccades were performed, and reliance on rereading, both of previously read text and regressions to previous words on the current line of text, was lower. It is possible that the pattern reflects that lower effort is used when building the meaning representation of the text ( 29, 39 ). Furthermore, no difference in these demands is found in total reading times towards the ends of texts, as meaning never becomes adequately constructed during reading, which also results in lower comprehension scores. Word skipping is an automatic part of reading ( 1, 7, 17, 50 ), but is also consciously affected by the type of reading, which was seen in the lower number of visits on words during skimming. Increased reading speed and fewer visits on words leading to lower comprehension agrees with Rayner et al. ( 37 ) in their critique of speedreading, where they argue that reading speed can only be increased while maintaining high comprehension by increasing language skill.

### Spell Checking

Trial durations were significantly longer, compared to regular reading. This means that people spent more time reading the texts before moving to the next one.

*Replication of previous research.* Spell checking was the only reading instruction where participants were not reading normally but rather searching for errors. This task effect was shown in lower comprehension scores, but more importantly, in significantly larger frequency effects compared to regular reading in first-pass reading times and total reading times. This replicates both Kaakinen and Hyönä ( 27 ) and Schotter et al. ( 43 ), and shows that participants are accessing the words to a larger extent than when reading regularly in order to determine whether spelling is correct or not. With infrequent words, i.e., words that the participants are not that familiar with, this process is more difficult, resulting in longer fixation durations. Significantly larger word length effects compared to regular reading were also found in first-pass reading times and total reading times, replicating Kaakinen and Hyönä ( 27 ). This further suggests that words are inspected, rather than read, during spell checking. Spell checking had significantly shorter saccade amplitudes, compared to regular reading. This replicates the results in Kaakinen and Hyönä ( 27 ). In Kaakinen and Hyönä ( 27 ), refixation probability was also found to be higher. In the current study, total number of visits increased significantly compared to regular reading, which can be considered similar to the finding by Kaakinen and Hyönä ( 27 ).

*Discussion of new findings.* Compared to regular reading, spell checking showed significantly longer first fixation durations and first-pass reading times. These early effects show that participants changed their early processing because of the task. Shorter saccade amplitudes combined with longer average fixation durations during the entire trial show that the reading pattern was much more deliberate, with higher amounts of processing during each fixation on a word. A significant difference in overall reading across the page was found, with regular and thorough reading showing a consistent decrease in total reading times per character and total number of visits, while spell checking showed a striking evenness across the page. As with skimming, it is possible that the pattern reflects that lower effort is used when building a meaning representation of the text ( 29, 39 ). While total reading times are longer, no decrease in these demands are found in total reading times towards the ends of texts, as meaning never becomes adequately constructed during reading, which also results in lower comprehension scores. A higher number of regressions and a higher proportion of vertical saccades were also found compared to regular reading, showing that the pattern of reading was very different. This further indicates that people use a different reading strategy when spell checking, increasing their processing and distributing it much more evenly across the page. It is plausible that a higher amount of activation is needed to correctly identify the spelling of the word, compared to identifying sufficient amounts of meaning to continue reading. The larger effects of both frequency and word length indicate that processing of long and infrequent words were increasingly difficult when correct spelling was needed. The increases in total reading times led to increased comprehension for thorough reading, but a decrease in comprehension was seen for spell checking. This suggests that global eye movements are highly dependent on task demands, and not sufficient when investigating learning ( 20 ). This is further suggested by previous research in multimedia learning showing only inconsistent links between eye movements and learning ( 9, 42, 46 ).

## Conclusions

Early effects found in eye-tracking research on single sentences were replicated in this study, namely the word frequency effect showing increasing first fixation durations on less frequent words, as well as the word length effect showing increasing first fixation durations on longer words. The “usual” sentence-level effects can be found, but conclusions about reading an entire page of text can also be drawn. Using paragraphs as stimuli in eye-tracking studies can therefore be seen as viable and very useful, and should encourage the field to move beyond the traditional sentence-level reading research (also see 25 ).

Compared to regular reading, all three types of reading investigated in this study were notably different. During thorough reading, longer total reading times and a higher number of visits on each word were found, along with higher comprehension scores. This suggests that the material had been read more thoroughly. With regards to the reading pattern over the entire page, similarities were found with regular reading, with total reading times and total number of visits steadily decreasing down the page. This suggests that earlier material is reread to a higher extent. People might need to recheck forgotten details because the context needs to be firmly established during initial reading, or simply because it is possible to reread that material but not possible to reread text that has not yet been seen. During skimming, longer saccades and shorter average fixation durations were combined with fewer visits on each individual word, along with lower comprehension scores. This is interpreted as more word skipping, less processing, and less rereading, respectively, and led to less thorough reading reflected in lower comprehension. Total reading times were also shorter and more evenly distributed across the entire page compared to regular reading. This suggests that reading was deliberately faster and more uniform. During spell checking, shorter saccades and longer average fixation durations were found, along with lower comprehension scores. This is interpreted as less word skipping, more processing, but that these factors do not signify thorough reading, as comprehension was drastically worse. Total reading times were longer and evenly distributed across the entire page. This suggests that reading was deliberately slower and more uniform, but that these results cannot be considered “more thorough” with respects to regular reading, as comprehension decreased.

### Ethics and Conflict of Interest

The author(s) declare(s) that the contents of the article are in agreement with the ethics described in http://biblio.unibe.ch/portale/elibrary/BOP/jemr/ethics.html and that there is no conflict of interest regarding the publication of this paper.

### Acknowledgements

This study was supported by funding from the Marcus and Amalia Wallenberg Foundation. We also thank the two anonymous reviewers for helpful comments on a previous version of this manuscript.

## Appendix 1. Example Stimuli Text in English (Including Line Breaks)

One of the many things that threaten animals and plants alike is the danger of being eaten. Some animals take recourse in camouflaging themselves, while others hide away. A number of them can fly away and others are so fast that they can flee from their enemies. Plants, which as is well known cannot run away, have developed other means to protect themselves. These include sharp spines and thorns or a tough skin. Other plants and also a number of animals protect themselves by poisons that they contain. These poisons need not necessarily be deadly. It is sufficient if they prevent other animals from eating the poisonous beings. Some animals even protect themselves by looking like others that contain poisonous substances. In the animal kingdom, a particularly striking color generally signals that the animal is inedible.
